# Trends in Opioid Prescribing and Dispensing by Veterinarians in Pennsylvania

**DOI:** 10.1001/jamanetworkopen.2018.6950

**Published:** 2019-01-11

**Authors:** Dana L. Clarke, Kenneth J. Drobatz, Chloe Korzekwa, Lewis S. Nelson, Jeanmarie Perrone

**Affiliations:** 1 Department of Clinical Sciences and Advanced Medicine, University of Pennsylvania School of Veterinary Medicine, Philadelphia; 2 currently anundergraduate student at Trinity College, University of Dublin, Dublin, Ireland; 3 Department of Emergency Medicine, Rutgers New Jersey Medical School, Newark; 4 Division of Medical Toxicology, Department of Emergency Medicine, Perelman School of Medicine, University of Pennsylvania, Philadelphia

## Abstract

**Importance:**

Veterinarians are a subset of opioid prescribers.

**Objective:**

To assess the quantity and trends in prescribing and dispensing of several different opioids in the past 11 years in a large veterinary hospital in Philadelphia, Pennsylvania.

**Design, Setting, and Participants:**

This cross-sectional study inventoried all opioid tablets and/or patches dispensed or prescribed by veterinarians practicing in a multispecialty academic veterinary teaching hospital in Philadelphia for small animals and species, such as rabbits, birds, and reptiles, from January 1, 2007, through December 31, 2017. Prescribing data were obtained from all veterinarians who wrote a prescription for opioids by reviewing detailed pharmacy records of controlled substances for the study period. Data included all opioids dispensed, or prescribed to animals (patients) undergoing evaluation at the center or being followed up as outpatients by the veterinarians in the hospital. Statewide veterinarian prescribing data were used for comparison. Data were analyzed from December 24, 2017, through May 15, 2018.

**Main Outcomes and Measures:**

The trend in administration and prescribing of 4 specific opioid analgesics (codeine sulfate, hydrocodone bitartrate, and tramadol hydrochloride tablets as well as fentanyl citrate patch) during the 11-year study period. The individual opioids were converted to morphine milligram equivalents (MME) for comparison.

**Results:**

The study included 134 veterinarians (70.9% women) with 366 468 patient visits. During the study period, the hospital veterinarians prescribed 1 051 836 tablets of tramadol, 97 547 tablets of hydrocodone, 38 939 tablets of codeine, and 3153 fentanyl patches to dogs (73.0%), cats (22.5%), and exotic animals (4.5%). Overall, MME use increased 41.2%, whereas visits increased by 12.8%. The comparison data for Pennsylvania revealed a predominance of hydrocodone use (688 340 tablets prescribed), although data were not available for comparison with tramadol because it is a Schedule IV drug.

**Conclusions and Relevance:**

Results of this study suggest that the large, increasing volume of opioids prescribed at 1 veterinary teaching hospital highlights concerns parallel to those about excessive opioid prescribing in humans. The extent to which these data may represent similar volumes of prescriptions from the general veterinary practices and hospitals across the United States is suggested by the accompanying Pennsylvania state data. These findings highlight an opportunity to assess the risk of veterinarian opioid prescriptions to safeguard public health.

## Key Points

**Question:**

What kind of opioids and how many are prescribed by veterinarians?

**Findings:**

In this cross-sectional inventory study of opioid prescribing by 134 veterinarians in a multidisciplinary acute care veterinary teaching hospital in Pennsylvania, a parallel trend of escalating opioid prescriptions and potency was found from 2007 through 2017. The substantial and increasing volume of opioids prescribed highlights analogous concerns about excessive opioid prescribing in humans.

**Meaning:**

Veterinarians prescribe a substantial amount of opioids, so prescribing practices of veterinarians merit further evaluation to safeguard public health.

## Teaser

This cross-sectional inventory study assesses the quantity and trends of several different opioids prescribed and dispensed for small animals and exotic species during the past 12 years in a large veterinary hospital in Philadelphia, Pennsylvania.

## Introduction

The United States is in the midst of an opioid crisis that has expanded from oral prescribed opioids to include heroin and highly lethal fentanyl analogues. Approximately 4 of 5 people who use heroin previously used prescription drugs.^1^ A major factor contributing to this epidemic is the overall increase in the ready availability of opioids. Patients with a history of opioid use disorder reported obtaining their opioids primarily for free from a friend or relative or from a physician.^2^ Although medical and dental health care professionals have been the major source of these opioids, the contribution of veterinary prescribing has not been quantitatively assessed.

Veterinarians and veterinary hospitals can be registered with the US Drug Enforcement Administration and in many states can administer, prescribe, stock, and dispense opioids, often without a reporting requirement to the state’s Prescription Drug Monitoring Program (PDMP).^3^ This situation may create a pathway that allows humans to covertly access opioids for diversion or misuse from their pets or other animals. In addition, leftover opioids from veterinary prescriptions can also result in diversion, misuse, abuse, or inadvertent toddler exposure. We investigated the potential volume of various opioids available through veterinary sources by inventorying the controlled substance records of a single veterinary hospital during an 11-year period that paralleled the rise of the opioid crisis.

## Methods

This cross-sectional study inventoried all opioid tablets and/or patches dispensed or prescribed by veterinarians practicing in a multispecialty academic veterinary hospital at the University of Pennsylvania School of Veterinary Medicine, Philadelphia, from January 1, 2007, through December 31, 2017. Data were analyzed from December 24, 2017, through May 15, 2018. This study was reviewed by the institutional review board of the University of Pennsylvania, which determined that it did not meet the criteria for human subject research. This study followed the Strengthening the Reporting of Observational Studies in Epidemiology (STROBE) reporting guideline.

Prescribing data were obtained by reviewing detailed, controlled substance pharmacy records in a single acute care veterinary referral hospital with 36 347 annual visits in fiscal year 2018 to describe trends in opioid prescribing and dispensing. Three of the most frequently used opioids (hydrocodone bitartrate, tramadol hydrochloride, and codeine sulfate) were tabulated, and fentanyl citrate was dispensed in patch form. All tablet strengths were included, and each opioid tablet was converted into morphine milligram equivalent (MME) amounts using standard conversion formulas.^4^ Only solid forms (pills and patches) were included; liquid formulations were omitted.

We used the Pennsylvania state veterinary opioid prescribing data in the US Drug Enforcement Administration Automation of Reports and Consolidated Orders System (ARCOS) for 2014 through 2017 to assess the magnitude of overall veterinary opioid prescribing in Pennsylvania and to compare the relative frequency and veterinary use of each opioid on a statewide level.^5^ However, tramadol (a Schedule IV drug) prescribing information was not available in ARCOS because only Schedule II and III drugs were included. Descriptive statistics were used.

## Results

In this acute care multidisciplinary veterinary hospital, any licensed veterinarian (intern, resident, or faculty) can use the hospital Drug Enforcement Administration number to order and prescribe opioids. In 2017 through 2018, 134 veterinarians (80 house staff and 54 faculty members; 39 men [29.1%] and 95 women [70.9%]) practiced. From 2007 through 2017, hospital visits increased from 29 899 to 33 730, for a total of 366 468. During the study period, the hospital veterinarians prescribed a total of 1 051 836 tablets of tramadol, 97 547 tablets of hydrocodone, 38 939 tablets of codeine, and 3153 fentanyl patches to dogs (73.0%), cats (22.5%), and exotic species such as rabbits, birds, and reptiles (4.5%). (Table) Although detailed records of new prescriptions compared with refills were not available, a manual evaluation of prescriptions in 2017 demonstrated that approximately 10% were refills. Overall, MME use increased 41.2% (Figure), whereas visits increased by 12.8% in the same period. The Pennsylvania ARCOS data revealed a predominance of hydrocodone use (688 340 tablets dispensed), although tramadol data were not available for comparison because it was under a different controlled substance schedule (Schedule IV).

**Table.** Comparison of Prescribed Opioids Between the Study Hospital and Pennsylvania ARCOS Database^a^

Opioid	No. of Tablets or Patches^b^	
	Study Hospital	ARCOS Database^c^
Tramadol hydrochloride tablets	1 051 836	Not included^d^
Hydrocodone bitartrate tablets	97 547	688 340
Codeine sulfate tablets	38 939	14 100
Fentanyl citrate patches	3153	23 110
Hydromorphone hydrochloride	Not used	171 100
Oxycodone hydrochloride	Not used	7600

Abbreviation: ARCOS, Automation of Reports and Consolidated Orders System.

^a^ Obtained from the University of Pennsylvania School of Veterinary Medicine, Philadelphia, from 2007 through 2017 and the Pennsylvania ARCOS database that tracks opioid delivery from manufacture to point of sale from 2014 through 2017.

^b^ Includes individual tablets of all strengths combined and individual fentanyl patches of all strengths combined.

^c^ A negligible fraction of opioid tablets are returned to the distributor and not dispensed.

^d^ Schedule IV compound is not tracked by ARCOS.

**Figure.**
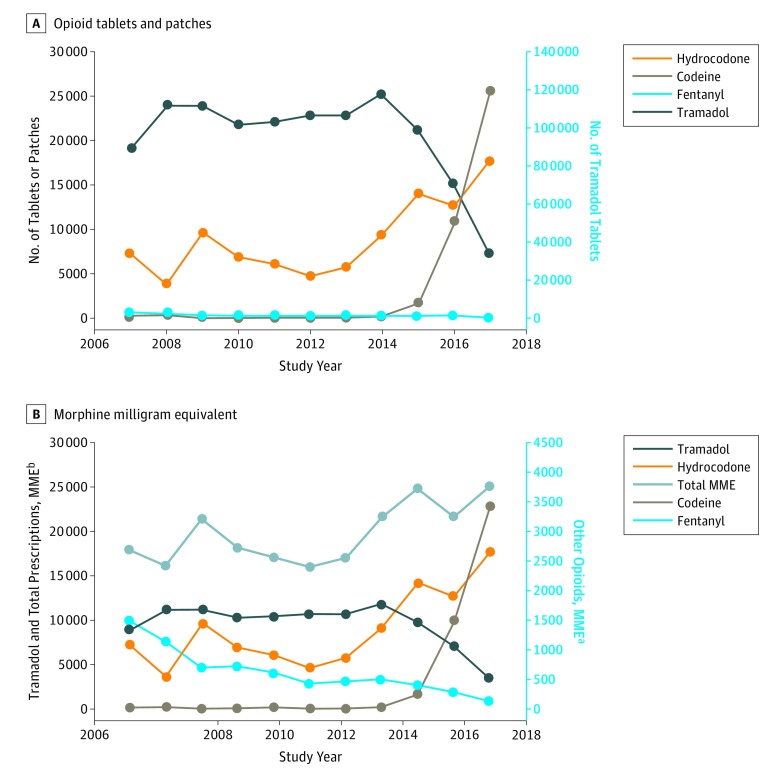
Changes in Opioid Dispensing and Prescribing in Tablet and Patch Forms and Morphine Milligram Equivalents (MMEs) During the Study Period Data are obtained from the veterinary hospital at the University of Pennsylvania School of Veterinary Medicine, Philadelphia, from 2007 through 2017. ^a^Includes hydrocodone bitartrate and codeine sulfate tablets as well as fentanyl citrate patches. ^b^Prescribed as tramadol hydrochloride.

## Discussion

Veterinarians administer, dispense, and prescribe substantial amounts of opioid analgesics for the management of pain and cough in animals. We report the opioid use practices of 1 hospital and 1 state for several years. The volume of opioids dispensed through veterinarian practices and hospitals in Pennsylvania may be projected to occur on a proportional basis in a subset of the estimated 190 million veterinarian visits (for cats and dogs) in the United States annually. Although we did not ascertain the indications for this opioid use, the specific opioids and trends in opioid prescribing at this acute care institution over time may reflect the practice pattern of specific veterinarians, a deliberate change in practice in the field, scheduling changes among the opioids, or shifting cost and availability of specific opioids.

Although we report the numbers of opioid tablets prescribed by veterinarians at 1 hospital during this study period, it can be reexamined in the context of overall annual hydrocodone prescriptions nationally. In 2015, IQVIA (formerly Quintiles and IMS Health, Inc) reported 5.7 billion tablets of hydrocodone tablets prescribed to humans.^6^ Although we have not tabulated national annual veterinary opioid prescribing, the 17 868 tablets of hydrocodone prescribed and dispensed by this hospital in 2017 is but a small fraction of overall hydrocodone use.

Overall, prescribing of tramadol tablets and fentanyl patches has decreased substantially in this population. Tramadol prescribing may have declined owing to the lack of efficacy in dogs, who constituted most of the animals evaluated despite some still undefined benefit in cats.^7^ Fentanyl patch prescribing may have declined owing to increasing awareness of the risks of fentanyl patches, including the quantity of leftover medication in the patch, risks to toddlers, the requirement for US Food and Drug Administration–mandated Risk Evaluation and Mitigation Strategies, and perhaps the public warning about fentanyl-related death.

Although extensive efforts have been made in educational campaigns about opioid misuse directed toward physicians and dentists, similar programs have not been replicated for veterinarians. The recent increased scrutiny of medical and dental opioid prescribing may have redirected some individuals to obtain opioids from veterinarians.^8^ In a recent survey,^9^ 73% of veterinarians reported little education about opioid misuse in their training. This lack of awareness may have led to an increased vulnerability to such fraud.^9^ Concerns about diversion and misuse are now being addressed among this latter group.^10^ In addition, the extensive use of opioids may leave unused pills in homes with pets, raising the risk for exploratory diversion by teenagers or unintentional exposure in toddlers.

Regulatory oversight and prescriber educational efforts analogous to those in medicine are needed in veterinary practice. Despite mandatory reporting to the PDMP for scheduled drugs for human use, only 20 states mandate that veterinarians report their opioid prescribing.^3^ Although logistically complicated to enact, a requirement to access the PDMP may provide valuable information for veterinarians and other health care professionals as well. Further exploration is warranted to examine opportunities to manage veterinary opioid prescribing to mitigate human consequences.

### Limitations

This study focused on detailed prescribing records for controlled substances at 1 acute care, multispecialty veterinary teaching hospital in Pennsylvania. The generalizability of these opioid prescribing rates may underestimate or overestimate opioid use by veterinarians in general practice depending on the potential higher acuity of patients evaluated at this institution. However, the Pennsylvania ARCOS veterinary data add some perspective about veterinary prescribing in the same state.

## Conclusions

Results of this study suggest that the large and increasing volume of opioids prescribed at 1 veterinary hospital in Pennsylvania highlights parallel concerns about excessive opioid prescribing in humans. The extent to which these data may represent similar volumes of prescriptions from the general veterinary practices and hospitals across the United States is suggested by the accompanying Pennsylvania state data. These findings highlight an opportunity to assess the risk associated with veterinarian opioid prescriptions and develop mitigation strategies, including expanding veterinary PDMP reporting nationally to safeguard public health.
