# Incorrect Number of Tramadol Tablets Prescribed

**DOI:** 10.1001/jamanetworkopen.2019.0772

**Published:** 2019-03-01

In the Original Investigation titled “Trends in Opioid Prescribing and Dispensing by Veterinarians in Pennsylvania,”^1^ published January 11, 2019, a decimal that was omitted in the editorial process led to an incorrect number of prescribed tramadol tablets reported in the Abstract, Results, and Table. The correct number of tramadol tablets is 1 051 836, which was correctly reflected in the Figure. This article was corrected online.^1^
